# Leveraging Dietary Interventions to Benefit Patients with Hematologic Malignancies and Clonal Hematopoiesis

**DOI:** 10.3390/nu18101562

**Published:** 2026-05-14

**Authors:** Camille Brzechffa, Angela G. Fleischman

**Affiliations:** 1 School of Medicine, University of California, Irvine, CA 92697, USA; cbrzechf@hs.uci.edu; 2 Division of Hematology/Oncology, UC Irvine Health, Irvine, CA 92697, USA; 3 Chao Family Comprehensive Cancer Center, University of California, Irvine, CA 92697, USA

**Keywords:** dietary intervention, Mediterranean diet, plant-based diet, gut microbiome, clonal hematopoiesis, CHIP, myeloproliferative neoplasm, multiple myeloma, acute leukemia, inflammation, obesity

## Abstract

Diet is a modifiable factor that influences multiple pathways relevant to hematologic malignancy, including systemic inflammation, immune cell activity, gut microbiota composition, and cancer cell metabolism. Translation of preclinical findings into clinical practice for hematologic malignancies remains nascent, although momentum is building to evaluate dietary interventions as a component of supportive and disease-modifying care. This review examines the mechanistic rationale for dietary interventions across the spectrum of clonal hematologic disorders and synthesizes current clinical evidence. Anti-inflammatory dietary patterns, particularly the Mediterranean diet, have demonstrated reductions in pro-inflammatory cytokines and may attenuate the inflammatory milieu that fuels clonal expansion. Obesity, which elevates the risk of developing hematologic malignancies and worsens clinical outcomes in diseases such as acute lymphoblastic leukemia (ALL) and acute myeloid leukemia, may be addressed through calorie-restricted, low-fat, or plant-based dietary strategies. Gut microbiota dysbiosis induced by chemotherapy represents another target, with high-fiber and plant-based diets showing promise in restoring microbial diversity and potentially enhancing treatment efficacy. Early-phase clinical trials in multiple myeloma, acute lymphoblastic leukemia, and myeloproliferative neoplasms have established feasibility and yielded preliminary signals warranting larger confirmatory studies. Larger, rigorously designed trials are needed to establish dietary interventions as legitimate therapeutic tools in the management of hematologic malignancies.

## 1. Introduction

It has long been recognized that diet and nutrition are influential factors affecting human health and functioning. Diet and nutrition are increasingly being explored as a means to augment current therapies, slow disease progression, and improve quality of life across a range of health conditions including cancer. Preclinical studies in mice [1,2] as well as systematic reviews [3,4,5] have demonstrated the benefits of nutritional supplements or dietary interventions in various cancers including head and neck cancer, breast cancer, and lung cancer in improving treatment efficacy and clinical outcomes post-treatment. Diet may modulate cancer via multiple mechanisms, including altering immune cell activity, changing gut microbiota composition, and manipulating cancer cell metabolism. Although preclinical data provide a compelling rationale that diet could similarly impact hematologic malignancies, clinical evaluation in this setting has lagged behind solid tumors and remains an emerging area of investigation. Caveats apply when extending findings from solid-tumor studies to hematologic disease, given fundamental differences in cell of origin, tumor microenvironment, and treatment exposures, but the solid-tumor literature establishes biological plausibility worth interrogating in hematologic settings. In this review, we discuss the rationale for dietary interventions in hematologic malignancies through three interconnected mechanisms: reduction of disease-driving inflammation, mitigation of obesity-associated risks, and restoration of gut microbiota homeostasis. We then examine how dietary interventions have been utilized in clinical trials involving patients with specific hematologic disorders and conclude with a discussion of the challenges and opportunities for future research in this field.

We conducted a narrative review of the English-language literature published from inception through March 2026, searching PubMed, Embase, and ClinicalTrials.gov using combinations of the terms diet, nutrition, dietary intervention, Mediterranean diet, plant-based diet, fiber, fasting, gut microbiome, inflammation, and obesity, paired with hematologic malignancy, leukemia, myeloproliferative neoplasm, myelodysplastic syndrome, multiple myeloma, lymphoma, and clonal hematopoiesis. We included preclinical studies, observational studies, and interventional human studies. We did not apply formal systematic-review methodology and acknowledge the potential for selection bias inherent to narrative synthesis.

## 2. Inflammation and Hematologic Malignancies

### 2.1. Hematologic Malignancies Are Fueled by Inflammation

Hematologic malignancies are characterized by their origin from the hematopoietic system and include diseases such as myeloproliferative neoplasms (MPN), myelodysplastic syndromes (MDS), lymphomas, multiple myeloma (MM), and leukemias. Despite their different cellular origins within the bone marrow, a common thread among many hematologic malignancies is their association with inflammation [6,7], although the dominant cytokines and the directionality of the inflammatory contribution differ across diseases. For instance, in patients with acute myeloid leukemia (AML), pro-inflammatory cytokine interleukin-1 (IL-1) elicits a profound expansion of myeloid progenitors while suppressing the growth of normal progenitors [8]. Furthermore, pro-inflammatory cytokines such as tumor necrosis factor-α (TNF-α) and interleukin-6 (IL-6) are commonly upregulated in patients with myeloid malignancies [9]. In the case of MPN, increased levels of pro-inflammatory cytokines and elevated white blood cell counts are positively correlated with increased disease progression [10]; TNF-α is required for expansion of mutant cells in mouse models of MPN [11], although whether this requirement extends to human disease remains incompletely established. This shared characteristic of inflammation among a diverse set of hematologic malignancies provides a unique opportunity to understand how dietary interventions, either alone or in tandem with current therapies, may reduce malignancy-associated inflammation.

Inflammation has also been identified as a key factor in driving the progression and expansion of clonal hematopoiesis of indeterminate potential (CHIP), a condition that affects a substantial portion of aging adults. CHIP refers to the presence of somatic mutations in hematopoietic cells at a variant allele frequency of at least 2% in individuals without cytopenia or an overt hematologic malignancy [12,13,14,15,16]. Individuals with CHIP have higher pro-inflammatory markers including TNF-α, monocyte chemoattractant protein 1 (MCP-1), and high-sensitivity C-reactive protein (hs-CRP) [17,18,19]. Commonly mutated CHIP genes include DNA methyltransferase 3A (*DNMT3A*), which catalyzes de novo methylation of cytosine at CpG sites, and ten eleven translocation 2 (*TET2*), an Fe(II)- and 2-oxoglutarate-dependent methylcytosine dioxygenase that oxidizes 5-methylcytosine to 5-hydroxymethylcytosine and thereby initiates active DNA demethylation; loss of function in ether gene leads to excessive inflammatory secretion [20,21]. In mouse models, *TET2* deficiency is associated with higher circulating levels of interleukin-1β (IL-1β) via activation of the NLRP3 inflammasome [22]. It has been proposed that CHIP promotes a vicious pro-inflammatory feedback loop; inflammation initially predisposes to the development of CHIP, provides mutant CHIP cells with a selective growth advantage, and further upregulates pro-inflammatory cytokine secretion [23].

The presence of CHIP significantly increases one’s risk for developing a hematologic malignancy, but most people with CHIP will not progress to an overt disease. As a result, the non-malignant consequences of CHIP, including atherosclerotic cardiovascular disease (CVD), represent a more significant burden at the population level. CHIP carriers are nearly twice as likely to experience coronary artery disease even after adjusting for known CVD risk factors such as smoking and high cholesterol [18,24]. Recently, it was reported that a high saturated fat diet based on animal fats enhanced IL-1β secretion, which thereby accelerated the clonal expansion of *TET2*-deficient HSCs and worsened atherosclerosis in low-density lipoprotein receptor-deficient mice that had undergone a partial bone marrow reconstitution with *TET*2-deficient cells [23]. These findings, derived in a partial bone marrow chimera model in mice, will require validation in human cohorts before clinical inference is appropriate, but they provide a mechanistic rationale for evaluating dietary interventions in human CHIP carriers at elevated cardiovascular risk.

### 2.2. Dietary Interventions to Lessen Inflammation

Several dietary patterns have been proposed to reduce chronic inflammation through overlapping but distinct mechanisms. Their composition, intended duration, proposed mechanism in hematologic disease, and clinical or preclinical application are summarized in Table 1.

An umbrella review of meta-analyses encompassing more than 12,800,000 participants demonstrated a protective association between Mediterranean diet adherence and cardiovascular disease, cancer, and diabetes [25], although the underlying meta-analyses included a mix of observational cohorts and randomized trials, and effect sizes were generally larger in observational data than in randomized comparisons. The PREDIMED trial showed that Mediterranean diet interventions decreased serum C-reactive protein, IL-6, and endothelial and monocyte adhesion molecules compared to a low-fat diet [26], with cardiovascular event reduction reported in the primary endpoint analysis [27]. A randomized trial demonstrated reductions in monocyte CD49d and CD40, markers of immune cell activation [28]. Mediterranean diets have similarly reduced inflammatory biomarkers and improved quality of life in rheumatoid arthritis and systemic lupus erythematosus [29,30].

Whole-food plant-based diets, defined by emphasis on whole grains, legumes, fruits, vegetables, nuts, and seeds with minimization or exclusion of animal products, provide a prescriptive structure that may be advantageous for trials seeking a precise dose-response between intake and biological response. Plant-based dietary interventions have shown early promise in multiple myeloma, where they are associated with greater gut microbial diversity and increased production of butyrate, a short-chain fatty acid with anti-inflammatory properties [31]. The Dietary Approaches to Stop Hypertension (DASH) diet, which emphasizes vegetables, fruits, whole grains, low-fat dairy, lean protein, nuts, and legumes while limiting sodium, added sugars, and saturated fat, shares an anti-inflammatory profile with the Mediterranean and plant-based patterns and is well-validated for cardiovascular risk reduction.

High-fiber diets exert complementary effects through the gut microbiome. Fermentation of dietary fiber by intestinal bacteria generates short-chain fatty acids, which suppress pro-inflammatory cytokine production and strengthen intestinal barrier integrity. Emerging evidence also links higher fiber intake to improved responses to immunotherapy [32]. Importantly, both plant-based and high-fiber dietary patterns confer well-established cardiovascular benefits. A meta-analysis of prospective cohort studies including more than 410,000 participants demonstrated that greater adherence to plant-based diets is associated with reduced cardiovascular mortality and incidence of cardiovascular disease [33]. Similarly, a systematic review and dose-response meta-analysis of 22 cohort studies found that each 7 g/day increase in dietary fiber intake is associated with a 9% reduction in the risk of both cardiovascular disease and coronary heart disease [34]. These effects are particularly relevant for individuals with CHIP, who have an elevated risk of atherosclerotic disease. Taken together, anti-inflammatory dietary strategies may have dual relevance in hematologic malignancies by modulating disease biology and reducing cardiovascular risk, and they warrant prospective evaluation in CHIP populations.

**Table 1:** Composition, timing, and hematologic application of the principal dietary patterns under investigation in clonal hematologic disease.

Diet	Defining Features	Approx. Macronutrient Distribution	Duration/Timing in Clinical Use	Proposed Mechanism in Heme Malignancy	Hematologic Application
Mediterranean	Extra-virgin olive oil as principal fat; ≥3 servings fruit/day; ≥2 servings vegetables/day; ≥3 servings legumes/wk; ≥3 servings fish/wk; nuts ≥ 3 servings/wk; moderate red wine; minimal red and processed meat	~35–40% fat (mostly MUFA), ~40–50% carb, ~15–20% protein	Sustained pattern; trials typically 12–15 wk to demonstrate adherence and biomarker change	Reduces CRP, IL-6, monocyte activation; supports butyrate-producing taxa; addresses cardiovascular risk in JAK2V617F MPN and CHIP	MPN: NUTRIENT [35], virtual MPN diet study [36]
Whole-food plant-based	Whole grains, legumes, fruits, vegetables, nuts, seeds; minimization or exclusion of animal products, refined grains, added sugars, and oils	~10–15% fat, ~70–75% carb (high fiber), ~10–15% protein	Sustained pattern; NUTRIVENTION used 12 wk catered meals plus 24 wk coaching	Increases fermentable fiber substrate, butyrate, microbial alpha diversity; improves insulin resistance and inflammatory monocyte subsets	MM precursors: NUTRIVENTION [37]; MM on lenalidomide: Shah 2022 [31]
Calorie-restricted	15–30% reduction in total energy intake from baseline while maintaining balanced macronutrient proportions; typically combined with macronutrient restriction in oncology trials	Macronutrient ratios preserved at population norms (~30% fat, ~50% carb, ~20% protein) but at reduced absolute intake	Most often deployed during a defined treatment window (e.g., the induction phase of chemotherapy)	Lowers circulating IGF-I and insulin; raises adiponectin; may increase chemotherapy sensitivity in lymphoid disease	B-ALL: IDEAL ^1^ trial [38]
Fasting-mimicking diet	5-day plant-based, low-protein, low-sugar regimen designed to mimic the metabolic effects of water-only fasting; refeeding between cycles	~1100 kcal day 1, ~700 kcal days 2–5; very low protein (~9–10% of energy) and very low sugar; predominantly plant fats and complex carbohydrates	Cyclical, typically administered for 5 days every 3–4 weeks, often timed to chemotherapy cycles	Reduces IGF-I, glucose, and leptin; differentially sensitizes tumor cells to cytotoxic therapy; reshapes antitumor immunity	Solid tumor data [39]; not yet tested in completed heme malignancy trials
Ketogenic	Very-low-carbohydrate, high-fat regimen sufficient to induce sustained nutritional ketosis; can be lard-based or plant-based depending on fat source	~70–80% fat, ~5–10% carb, ~15–20% protein	Used as a sustained pattern or in defined treatment windows; preclinical AML data used 3 wk dosing around FLT3 inhibition	Shifts host and tumor lipid metabolism, increases ketone bodies and PUFA ^2^:MUFA ^3^ ratio, modulates FLT3 signaling and lipid biosynthesis to enhance targeted therapy	FLT3-ITD AML preclinical [40]

^1^ Improving Diet and Exercise in ALL. ^2^ Polyunsaturated fatty acid. ^3^ Monounsaturated fatty acid.

## 3. Dietary Interventions to Mitigate Obesity-Associated Risks Within Hematologic Malignancies

### 3.1. Obesity Increases Risk for the Development of Hematologic Malignancies

Over the last several decades, the prevalence of obesity in the United States has increased. In this review, we define obesity as determined by the World Health Organization categorization as a body mass index (BMI) ≥ 30.0 kg/m^2^. It is important to note, however, that while BMI is a practical clinical marker of obesity, it has limitations due to its inability to distinguish adipose distribution. Adipose tissue is a metabolically active endocrine organ that secretes pro-inflammatory cytokines, including TNF-α and IL-6 [41,42], and visceral adipose tissue is substantially more inflammatory than subcutaneous fat, releasing IL-6 directly into the portal circulation and contributing to systemic inflammation independently of BMI [43,44]. Because BMI cannot capture this depot-specific inflammatory burden, measures such as waist circumference and waist-to-hip ratio may more accurately reflect the metabolically relevant adiposity that drives disease risk [45].

Recent evidence has established an association between obesity and an increased risk of hematologic malignancies. In a population-based case–control study of patients diagnosed with either AML or MDS, obesity, as defined by BMI, two years prior to diagnosis was associated with AML in males and females [46]. A meta-analysis of nine prospective cohort studies found summary relative risks of leukemia of 1.14 (95% confidence interval (CI), 1.03–1.25) for overweight individuals (BMI 25–30 kg/m^2^) and 1.39 (95% CI, 1.25–1.54) for obese (BMI > 30 kg/m^2^) individuals [47]. When stratified by subtype across four studies, the relative risk ratios associated with obesity were 1.25 for chronic lymphocytic leukemia, 1.65 for ALL, 1.52 for AML, and 1.26 for chronic myeloid leukemia (CML) [47].

Obesity has also been associated with MPN, including polycythemia vera (PV), essential thrombocythemia (ET), and primary myelofibrosis (PMF). In a nationwide population-based cohort study in Israel, 443 Israeli adolescents out of 2,516,256 study population developed MPN. Obesity, which was defined as a BMI ≥ 95th percentile, in adolescence significantly predicted increased risk of MPN with a hazard ratio of 1.81 (CI, 1.13–2.92) [48]. Conversely, Rolles et al. presented data at the 2024 American Society of Hematology Annual Meeting suggesting that obesity may actually confer a protective effect using mouse models [49]. The relationship between obesity and MPN is further complicated by the fact that JAK inhibitors, a cornerstone of MPN treatment, carry a notable side effect of increased appetite and weight gain [50]. These apparently contradictory findings can be reconciled in part. The Israeli cohort measured BMI in adolescence among individuals who later acquired JAK2V617F or related mutations as adults, capturing the chronic-inflammation hypothesis of obesity as a clonal expansion driver. The Rolles murine data come from a constitutive JAK2V617F-knock-in model in which the mutant clone is established before the dietary perturbation, a setting that may favor different metabolic dynamics. JAK inhibitor-induced weight gain further complicates clinical observation by introducing reverse causation. Adipose depot, sex, and disease stage are likely additional modifiers. Reconciling these data will require prospective human studies that measure waist circumference and visceral adipose tissue rather than BMI alone.

Obesity can influence the progression of premalignant conditions. For example, monoclonal gammopathy of undetermined significance (MGUS), a premalignant state to multiple myeloma, was associated with a BMI > 30 kg/m^2^ after adjusting for age, sex, education, and income (95% CI, 1.21–2.47) [51]. Interestingly, in the same cohort, physical activity was found to be inversely associated with MGUS. Physical activity influences the same cytokine and metabolic pathways that are dysfunctional in obesity [52]. More recently, the population-based iStopMM screening study (*n* = 27,217 with food-frequency questionnaire data, 1020 with MGUS) found no overall association between dietary patterns or specific dietary components and MGUS prevalence, though high dairy consumption was associated with increased odds of IgA MGUS specifically (OR 2.01, 95% CI 1.16–3.65) [53]. These findings temper the expectation that broad dietary modification will substantially reduce MGUS risk and instead point toward subtype-specific dietary effects worth further study. Given these associations, it uncovers a chance to potentially reduce risk associations with premalignant states of hematologic neoplasms by modifying risk factors associated with obesity via dietary interventions and/or increasing physical activity.

CHIP expansion is also exacerbated by obesity. In a UK biobank cohort of 47,466 individuals with known CHIP mutations, it was found that CHIP was present in 5.8% of the study population and was associated with a significant increase in waist-to-hip ratio (WHR), another measure of obesity. Furthermore, in an obesity mouse model, Pasupuleti and colleagues observed that obesity increased the expansion of *TET2^−^/^−^,* a common CHIP mutant, in preleukemic stem and progenitor cells [54]. These findings were consistent with observations in a cohort of 8709 postmenopausal women enrolled in the Women’s Health Initiative, whereas an obese BMI was associated with the presence of CHIP mutations [55]. This implies that dysfunctional metabolic activity and the pro-inflammatory conditions induced by obesity may accelerate aberrant clonal hematopoiesis and the expansion of CHIP mutations [56].

### 3.2. Mechanisms by Which Obesity Drives Hematologic Malignancies

The biologic mechanism for the emerging observations of associations between obesity and the risk of developing hematologic malignancies remains unclear and is an area that necessitates future research. However, one potential mechanism suggests that the metabolic consequences of obesity, such as insulin resistance, promote tumorigenesis via the increase in bioavailable insulin-like growth factor-I (IGF-I) [57]. In the case of hematologic neoplasms, both normal and neoplastic hematopoietic cell lines express IGF receptors. Furthermore, IGF activation is mitogenic for cell lines of myeloid and lymphoid leukemias and has been described as an upregulated and prevalent signaling pathway in myelodysplastic syndromes [58]. One potential dietary intervention that may be beneficial in addressing both the metabolic risks of obesity and progression of hematologic neoplasms is a calorie-restricted diet. Effective calorie restriction is achieved by reducing energy intake by 15–30% while also maintaining balanced proportion of macronutrients. Though it is unknown whether calorie-restricted dietary interventions may benefit patients with hematologic neoplasms, it is thought that calorie-restricted diets offer a dual advantage of helping induce weight loss as well as inhibiting the IGF-I signaling [1,39,59]. Altering the IGF-I signaling transduction pathway may help attenuate aberrant hematopoietic cell line expansion. It is important to note, however, that established and standardized nutritional recommendations for cancer care and treatment may conflict with the reduction in protein intake or weight loss associated with calorie-restricted diets.

It is well established that obesity is accompanied by dysregulated chronic low-grade inflammation [60]. In the case of MPN and CHIP, an obesity-induced pro-inflammatory environment may further promote dysfunctional clonal hematopoiesis. This may further be compounded by diet quality as high saturated diets can further trigger systemic inflammation within the innate immune system [61]. While preliminary data exists regarding the potential of anti-inflammatory diets as a tool in care for hematologic malignancies like MPN, a cross-sectional study among 1329 MPN patients found that reported consumption of fast food and refined sugar was associated with increased total symptom burden scores [62]. By reducing obesity-induced inflammation or by implementing a dietary intervention to reduce inflammation, this could also serve as alternative adjunctive treatment to reduce inflammation in hematologic malignancies [63]. There is a great need to further investigate the role of anti-inflammatory diets such as the Mediterranean diet in reducing the progression or symptom burden of MPN or in other hematologic malignancies as discussed earlier.

### 3.3. Obesity Influences Outcomes for Patients with Hematologic Malignancies

Obesity is not only associated with the development of hematologic malignancies but may also influence clinical treatment outcomes and increase mortality risk. In children with acute lymphoblastic leukemia (ALL), obesity has been linked to higher rates of relapse, lower survival, and greater susceptibility to treatment-related toxicities [64,65,66,67,68,69]. In a study of 1443 children with ALL, children with BMIs ≥ 30 kg/m^2^ had a higher incidence rate ratio for severe toxic events, liver and kidney failures, bleeding, abdominal complications, unexpected severe adverse reactions, and hyperlipidemia compared to healthy weight children (17 to <25 kg/m^2^) [66]. The study concluded that these observations could be attributed to the overtreatment of obese patients due to differences in pharmacokinetics dosing, leading to treatment-related toxicities.

In AML, the relationship between obesity and clinical outcomes is less straightforward, with studies reaching inconsistent conclusions. Notably, a study at the University of Nebraska Medical Center found that obesity and a BMI ≥ 30 kg/m^2^ was associated with worse overall survival in adult patients with AML compared to their normal weight counterparts [69,70]. Additionally, a prospective study by the American Cancer Society identified a significantly elevated mortality risk ratio for non-Hodgkin’s lymphoma and myeloma, with a dose-response trend of increasing mortality across higher BMI categories [71]. Taken together, these findings suggest that both children and adults with hematologic malignancies who are living with obesity may benefit from targeted dietary and physical activity interventions aimed at reducing treatment-related toxicities and improving survival.

Body habitus likely also influences symptom burden in MPN, a disease in which validated scoring tools exist and symptom reduction is a primary treatment objective. In a combined analysis of two cross-sectional surveys with MPN patients, the Danish Population-based study, and the International Fatigue Study, a U-shaped association between BMI and total symptom burden was observed with higher mean scores among underweight and obese patients compared to those of normal weight [72]. Modifying BMI and implementing lifestyle interventions therefore may also provide meaningful relief for MPN patients with high symptom burdens. Notably, JAK inhibitors can increase hunger through JAK2 blockade, which plays an important role in leptin-mediated satiety signaling [50]. This introduces a therapeutic paradox: while JAK inhibitors are prescribed primarily to alleviate symptoms, any resulting weight gain may counteract that benefit by independently worsening the clinical picture.

### 3.4. Dietary Interventions for Obesity and Hematologic Malignancies

It is important to recognize that dietary choices may also influence clonal hematopoietic disorders through pathways that are independent of their effects on body weight. Diets high in saturated fats or cholesterol can induce and exacerbate obesity [73], and adherence to Western dietary patterns, categorized as high in saturated fats and cholesterol, has been associated with an increased risk of CLL [74]. Recent preclinical work using the Eµ-TCL1 murine model of CLL further demonstrated that a high-fat, high-carbohydrate Western diet significantly shortened survival and accelerated splenic disease involvement compared with standard chow, accompanied by reduced gut microbial alpha diversity and altered community composition [75]. A retrospective cohort study using data from the UK biobank analyzed whole-exome sequencing data and survey-based information on health-associated behaviors to understand the connections between CHIP and atherosclerotic cardiovascular disease. Diet quality was categorized either into unhealthy, intermediate, or healthy based on patients’ self-reported intake of healthy elements (fruits and vegetables) or unhealthy elements (red meat, processed foods, added salt). They identified that CHIP mutations in genes such as *TET2*, *DNMT3A*, and *ASXL1* were present in 162 of 2271 participants (7.1%) with an unhealthy diet, 2177 of 38,552 participants (5.7%) with an intermediate diet, and 168 of 3288 participants (5.1%) with a healthy diet [76]. This suggests that unadjusted CHIP prevalence progressively decreases with healthier diet quality. This progressive decrease in unadjusted CHIP prevalence with healthier diet quality suggests that dietary composition itself, apart from its contribution to obesity, may modulate clonal hematopoiesis. This cross-sectional finding suggests that healthier diet quality is associated with lower CHIP prevalence, but it cannot establish that dietary improvement reduces CHIP, since reverse causation and unmeasured confounders are not excluded. Disentangling these independent effects of diet from the metabolic consequences of obesity, including chronic systemic inflammation, insulin resistance, and adipokine dysregulation, remains a significant challenge but an important area for future investigation.

Nonetheless, dietary interventions that address obesity directly also hold promise for patients with hematologic malignancies. Low-fat and low-carbohydrate diets have emerged as interventions for obesity management [77]. In adolescents with obesity and non-alcoholic fatty liver disease, moderate carbohydrate restriction (<25% of energy over 8 weeks, *n* = 32) produced significant improvements in insulin resistance, weight-related outcomes, and body composition compared with a reduced-fat diet [78]. Beyond their effects on weight, low-fat diets have been associated with reduced risk of liver cancer, decreased pancreatic growth, and lower breast cancer mortality [79,80,81,82,83]. In a murine model of leukemia, mice fed a low-fat diet supplemented with 1.5% *n*-3 fatty acid docosahexaenoic acid (DHA), in combination with arabinosylcytosine (AraC) chemotherapy, increased the survival time of BDF1 mice bearing L1210 cell-induced leukemia [82,83]. These murine findings provide preclinical proof of concept but have not been replicated in human leukemia trials and should not be extrapolated directly to clinical practice. Whether low-fat and low-carbohydrate diets can meaningfully reduce the risk of hematologic malignancy progression, and whether any such benefit is driven by weight reduction, by direct effects of macronutrient composition, or both, will require dedicated prospective trials.

More recent preclinical evidence has expanded the dietary repertoire under investigation in AML. Goupille et al. demonstrated in FLT3-mutated AML mouse models that a lipid-rich ketogenic diet enhanced the efficacy of FLT3 inhibition, reducing engraftment and tumor burden approximately twofold and rewiring tumor metabolism toward fatty acid oxidation [40]. The translational implications remain to be tested in human FLT3-mutant AML, but the data establish ketogenic dietary interventions as a candidate adjunct to targeted therapy worth clinical evaluation.

## 4. The Gut Microbiome as a Mediator of Dietary Effects in Hematologic Malignancies

Beyond its effects on obesity and systemic inflammation, diet may influence hematologic malignancies through modulation of the gut microbiome. Poor dietary patterns can disrupt microbial composition, compromise intestinal barrier integrity, and promote systemic inflammation that drives clonal hematopoiesis and malignant progression, while healthful diets cultivate a diverse, anti-inflammatory microbial community that may protect against these processes.

### 4.1. Gut Dysbiosis Promotes Inflammation and Drives Clonal Hematopoiesis

A healthy gut microbiome maintains intestinal barrier function in part through the production of short-chain fatty acids (SCFAs), particularly butyrate, which serves as the primary energy source for colonocytes and strengthens epithelial tight junctions [84]. The principal butyrate-producing taxa in the healthy human colon include *Faecalibacterium prausnitzii*, *Roseburia* species, and *Eubacterium rectale*; their relative depletion is a consistent feature of dysbiotic states and has been documented in inflammatory bowel disease, obesity, and several hematologic malignancies. When barrier integrity is compromised, microbial components such as lipopolysaccharide (LPS) translocate into the systemic circulation, triggering inflammatory cascades. This mechanism has been demonstrated principally in murine systems, and its quantitative contribution to human CHIP progression is still being defined. Meisel and colleagues demonstrated that disruption of the intestinal barrier led to bacterial translocation and elevated IL-6 production, which in turn induced preleukemic myeloproliferation in *TET2*-deficient mice [85]. More recently, Agarwal and colleagues identified that aging-associated increases in gut permeability allow ADP-heptose, a metabolite specific to Gram-negative bacteria, to enter the circulation and drive expansion of *DNMT3A*-mutant hematopoietic stem cells via the ALPK1-NF-κB signaling axis [86]. These findings establish a direct mechanistic link between gut barrier dysfunction, microbial metabolites, and CHIP progression in murine models, with extension to human disease still pending.

Dietary composition can further modulate this axis. Lyon and colleagues recently demonstrated that high vitamin B12 supplementation, a component abundant in animal-based diets, induced gut dysbiosis, reduced SCFA-producing bacteria, and enhanced myelopoiesis and inflammatory cytokine production in a *TET2*-deficient model of clonal hematopoiesis. Critically, these effects were reversible upon oral supplementation with butyrate, suggesting that restoring SCFA production can counteract diet-induced CHIP acceleration [87]. These observations come from a single preclinical study published as a preprint and have not yet been replicated or extended to human CHIP carriers. They should be interpreted as hypothesis-generating rather than as a basis for clinical recommendation regarding B12 intake.

### 4.2. Dietary Fiber, Butyrate, and Gut Barrier Function

Dietary fiber is the principal substrate for microbial butyrate production, and fiber intake is therefore a key determinant of gut barrier integrity. Butyrate strengthens the epithelial barrier through multiple mechanisms: it serves as the primary fuel for colonocyte oxidative metabolism, stabilizes hypoxia-inducible factor-1 (HIF-1) to upregulate tight junction proteins and mucin production, promotes regulatory T-cell differentiation, and suppresses NF-κB-mediated inflammation [84,88]. When dietary fiber is insufficient, glycan-consuming microbiota degrade the protective mucus layer as an alternative food source, further compromising barrier function [89]. In the murine model, fiber-deficient diets selectively favor mucin-degrading taxa such as *Akkermansia muciniphila* and *Bacteroides caccae*, with consequent thinning of the colonic mucus layer and increased pathogen susceptibility. This creates a vicious cycle in which low fiber intake leads to reduced butyrate production, weakened barrier integrity, increased translocation of inflammatory microbial products, and enhanced conditions for clonal expansion of mutant hematopoietic cells.

Clinical evidence supports the importance of fiber intake in cancer outcomes. In 128 patients with metastatic melanoma, Spencer and colleagues demonstrated that higher dietary fiber intake improved response to immune checkpoint inhibitors and decreased risk of disease progression [32]. In their accompanying mouse model, a high-fiber diet significantly delayed tumor growth with checkpoint inhibitor treatment and produced a distinct gut microbiome profile. Importantly, these findings were microbiome-dependent, as they could not be recapitulated in germ-free mice. These observations suggest that dietary fiber’s anti-cancer effects operate at least in part through gut microbiome modulation, though the human cohort was modest in size and fiber intake was assessed by self-report, both factors that limit the strength of inference.

### 4.3. Dietary Micronutrients with Disease-Specific Relevance

Dietary inputs can be both beneficial and detrimental in clonal hematologic disease, and several micronutrients have specific mechanistic relevance. Vitamin C is of particular interest in *TET2*-mutant clonal hematopoiesis. As a cofactor for Fe(II)- and 2-oxoglutarate-dependent dioxygenases, ascorbate facilitates the catalytic cycle of *TET2* and partially restores 5-hydroxymethylcytosine formation in *TET2*-deficient hematopoietic progenitors. Cimmino and colleagues showed in a reversible RNAi mouse model that vitamin C treatment mimics *TET2* restoration, suppresses leukemic colony formation, and slows leukemia progression in patient-derived xenografts [90]. Subsequent work by Guan and colleagues demonstrated that the cellular effect of ascorbate on *TET2* is context dependent and modulated by acetylation state and the specific *TET2* mutation, suggesting that pharmacologic combinations with HDAC inhibitors or sirtuin activators may be needed to achieve consistent effects [91]. Vitamin C and vitamin D deficiencies are common in patients with AML and other hematologic malignancies, and replacement may have a role both in supporting gut barrier integrity and in enzymatic restoration of *TET2* activity. By contrast, high-dose pharmacologic antioxidant supplementation during cytotoxic therapy raises a different concern. Many chemotherapeutic and radiotherapeutic mechanisms depend on the generation of reactive oxygen species, and there is theoretical and limited empirical concern that high-dose antioxidant supplementation may blunt cytotoxic efficacy [92]. Randomized trial evidence is mixed and underpowered, and it is important to distinguish dietary antioxidant intake from whole food sources, which is generally compatible with anti-inflammatory dietary patterns, from high-dose isolated supplementation taken during active treatment, where caution remains warranted. Folate also merits attention. Periconceptional dietary folate intake has been associated with reduced risk of pediatric ALL through modulation of early DNA methylation patterns, with effect direction varying by folate source [93]. Together, these examples reinforce that dietary effects on clonal hematologic disease are mutation-, context-, and treatment-specific, and that not all dietary inputs labeled healthful are uniformly beneficial in every clinical setting.

### 4.4. Chemotherapy and Antibiotics as Disruptors of the Gut Microbiome

Patients with hematologic malignancies face compounding threats to their gut microbiome. Cytotoxic chemotherapy, the standard treatment for most hematologic neoplasms, is associated with loss of microbial diversity, which has been linked to poor clinical outcomes including increased infections and higher mortality [83]. Reductions in gut microbiome diversity during chemotherapy have been observed in pediatric patients with acute lymphoblastic leukemia and in patients with acute myeloid leukemia [94].

This population is further vulnerable to gut dysbiosis because of extensive antibiotic exposure. Over 80% of patients with hematologic malignancies develop febrile neutropenia during chemotherapy, necessitating empiric broad-spectrum antibiotics [95]. Many patients also receive prophylactic fluoroquinolones during periods of neutropenia. Broad-spectrum beta-lactam antibiotics used for neutropenic fever have been shown to significantly reduce gut microbial alpha diversity in patients with hematologic malignancies [96]. The consequences of antibiotic-induced dysbiosis extend beyond infection risk. In patients with non-Hodgkin lymphoma, Pflug and colleagues found that anti-Gram-positive antibiotic exposure during platinum-based chemotherapy was associated with reduced chemotherapy response and overall survival, suggesting that specific gut bacteria may be necessary for maximum treatment efficacy [97]. This association is subject to confounding by indication, since patients receiving anti-Gram-positive antibiotics are by definition those with infectious complications, who tend to have higher disease burden, more intensive prior therapy, and worse baseline performance status. Whether antibiotic-induced microbiome disruption is itself causal, or marks a sicker patient population, remains to be resolved by prospective studies.

### 4.5. Dietary Strategies to Restore and Maintain a Healthy Microbiome: Prebiotics and Probiotics

Addressing gut dysbiosis in patients with hematologic malignancies may require a two-pronged approach encompassing both prebiotics and probiotics. Prebiotics are fermentable dietary fibers and substrates that selectively nourish beneficial gut bacteria. By promoting the growth of butyrate-producing species such as *Faecalibacterium prausnitzii* and *Roseburia*, prebiotic-rich diets can increase SCFA production, reinforce barrier function, and create an anti-inflammatory intestinal environment. Plant-based diets, which are inherently rich in fermentable fibers, may be particularly beneficial in this regard; as described in the clinical evidence below, plant-based dietary interventions have been associated with higher stool butyrate concentrations and greater microbial alpha diversity in patients with multiple myeloma [98].

Probiotics, defined as live microorganisms that confer health benefits when administered in adequate amounts, offer a complementary strategy by directly introducing beneficial bacterial species into the gut. However, establishing beneficial bacteria alone may be insufficient without the dietary substrates needed to sustain them. This highlights the importance of combining probiotic supplementation with prebiotic-rich dietary patterns to both seed and feed a healthy microbiome. Mediterranean and plant-based diets may achieve this synergy naturally, as they are rich in diverse fermentable fibers that support a broad range of beneficial microbial communities. For instance, in a murine model of colorectal cancer, tumor incidence was reduced in mice fed a Mediterranean diet mix compared to a low-fat or Western-style diet, and the Mediterranean diet improved chemotherapy-induced dysbiosis [99].

Targeting the gut microbiome through dietary interventions in patients with hematologic malignancies represents a promising strategy to maximize the efficacy of current treatments, mitigate treatment-related toxicities, and restore gut homeostasis. Given the vulnerability of this patient population to dysbiosis from both chemotherapy and antibiotic exposure, integrating microbiome-supportive dietary strategies into clinical care warrants further investigation through rigorously designed clinical trials.

## 5. Completed and Ongoing Dietary Interventions in Heme Malignancies

Completed and ongoing dietary intervention studies in hematologic malignancies are summarized in Table 2 and discussed in detail below.

### 5.1. Multiple Myeloma

Multiple myeloma (MM) is the second most common hematologic malignancy and is characterized by the aberrant proliferation of plasma cells. While current treatments for MM aim to improve longevity for patients, there is recent evidence that modifiable lifestyle factors such as dietary interventions and physical activity may also contribute to increasing quality of life [101]. Following therapy, the best predictor of survival for patients with MM continues to be sustained minimal residual disease (MRD) negativity [102]. To investigate whether a plant-based dietary intervention may have an association with sustained MRD negativity in patients with MM, Shah and colleagues assessed patients with MM on lenalidomide maintenance and collected stool samples to examine both the gut microbiome composition as well as the concentrations of butyrate, a short-chain fatty acid (SCFA) produced by gut microbes. After three months on a plant-based diet, sustained MRD negativity was associated with higher stool butyrate concentration, butyrate producers, and alpha diversity in the gut microbiome [31]. Further, consumption of dietary proteins from seafood and plants was correlated with higher butyrate concentrations at three months and sustained MRD negativity [31]. These results are consistent with the hypothesis that plant-based diets promote higher gut microbial diversity and increased SCFA production, which may benefit patients with multiple myeloma. The single-arm design and modest sample size preclude causal inference, and confirmation requires randomized controlled comparison. Dietary interventions may also provide protective effects such as reducing MM risk with increased consumption of vegetables or increased intake of whole grains and fiber [103,104].

The NUTRIVENTION trial reported by Shah and colleagues in 2026 has now provided the most rigorous evaluation of dietary intervention in myeloma precursor disease to date [37]. In this single-arm trial, 23 participants with myeloma precursor states (MGUS or smoldering myeloma) and elevated BMI received a high-fiber, whole-food, plant-based diet (catered meals for 12 weeks plus dietary coaching for 24 weeks). The intervention was feasible and improved quality of life along with metabolic (BMI, insulin resistance), microbiome (alpha diversity, butyrate-producer abundance), and immune (inflammatory cytokines, monocyte subsets) markers. Two participants experienced improvement in disease trajectory, and disease was stable in the remainder over the study period. In a parallel Vk*MYC mouse model of myeloma precursor disease, a high-fiber diet delayed disease progression independently of caloric restriction or weight change, an effect mediated by SCFA production and reinvigorated antitumor immunity. These data substantially strengthen the rationale for further randomized testing of high-fiber, plant-based dietary intervention in myeloma precursor states. Complementary descriptive work from the same research community has characterized dietary patterns among individuals with plasma cell disorders and identified opportunities for targeted nutritional intervention [105].

### 5.2. Acute Lymphoblastic Leukemia

Acute lymphoblastic leukemia (ALL) is one of the most common pediatric cancers [106]. Pediatric patients with ALL are especially at high risk for developing obesity [107]. Additionally, the early presence and diagnosis of obesity may contribute to chronic disease burden among pediatric ALL survivors. This presents an opportunity to understand how lifestyle interventions including dietary interventions may benefit pediatric patients with ALL. In a study of 13 pediatric patients diagnosed with ALL and their families from 2014 to 2016, lifestyle intervention feasibility over a 3-month period was assessed and included sessions targeting positive parenting styles, healthy eating practices, and increasing physical activity. Among the thirteen children enrolled, a trend of overall diet quality increased by 3.14 as measured by the Healthy Eating Index-2015 total score, along with decreased consumption of added sugars, and decreased percentage of consumption of potatoes [100]. Reductions in added sugar consumption reflect improvements in dietary quality, although the study did not measure cardiometabolic biomarkers and we cannot infer cardiovascular benefit from these intake changes alone. Importantly, this study highlighted the importance of parental education as parents reported a reduced “pressure to eat” feeding practice for their children by the end of the 3-month intervention. This finding is important as parents of children diagnosed with cancer have reported more permissive parenting styles following their child’s diagnosis including allowing the child to eat any foods that they may crave [108]. However, the study did not find any significant changes in the children’s level of physical activity or weight status.

In another dietary and exercise intervention, known as the Improving Diet and Exercise in ALL (IDEAL) trial, the authors were interested in studying whether a caloric deficit and macronutrient restricted diet would also improve chemosensitivity in obese or overweight patients with B-cell acute lymphoblastic leukemia (B-ALL) as measured by minimal residual disease status. The IDEAL trial included 40 patients with newly diagnosed high-risk B-ALL. The IDEAL intervention had no effect on reducing fat mass. However, the intervention did significantly reduce minimal residual disease risk, increased circulating adiponectin, and reduced insulin resistance [38]. The IDEAL trial used a nonrandomized historical control for comparison and a modest sample size, limitations that should be borne in mind when interpreting the MRD signal. The conclusions of this study emphasize a greater need for larger clinical trials and basic scientific research to address how lifestyle modifications could improve outcomes for patients with B-ALL.

Beyond intervention, dietary epidemiology has informed prevention research in pediatric ALL. Nickels and colleagues recently reported that periconceptional folate intake influences the DNA methylome of ALL diagnosed in the offspring, with concordant directions of effect between folate-associated and lymphoblast-associated differentially methylated regions [93]. These findings support a mechanistic role for dietary folate, particularly from natural food sources, in early epigenetic programming relevant to ALL risk.

### 5.3. Myeloproliferative Neoplasms

Myeloproliferative neoplasms (MPN) occupy a unique position at the intersection of heme malignancy, cardiovascular disease, and chronic inflammation. JAK2V617F is one of the most pro-atherogenic CHIP mutations, and while NCCN guidelines place cardiovascular risk factor management as the primary treatment strategy, no specific dietary guidelines are provided. Patients with MPN experience significant symptom burden including fatigue, pruritus, night sweats, and cardiovascular complications, all of which impair quality of life [109,110]. Given these challenges, we hypothesized that diet, and the Mediterranean diet in particular, could serve as a low-risk therapeutic approach in MPN.

We tested this in the NUTRIENT trial, a randomized phase I pilot study in which 28 MPN patients were assigned to either a Mediterranean diet or standard U.S. Dietary Guidelines, both with registered dietitian counseling [35]. We demonstrated that MPN patients can readily adopt a Mediterranean eating pattern, with approximately 80% of the Mediterranean group maintaining adequate adherence throughout the active intervention. Importantly, 53% of the Mediterranean group achieved a greater than 50% reduction in MPN-SAF Total Symptom Score by study end compared to 31% in the standard diet group, and we identified a significant correlation between improvement in Mediterranean diet adherence and symptom reduction across the entire cohort (week 9: r = −0.52, *p* = 0.007; week 12: r = −0.49, *p* = 0.012). As a phase I pilot with a primary endpoint of feasibility, NUTRIENT was not powered to establish efficacy, and the symptom-burden findings should be interpreted as hypothesis-generating. In parallel, we characterized the gut microbiome using shotgun metagenomics and found significant differences between MPN subtypes, with myelofibrosis patients exhibiting the most dysbiotic profiles, including reduced *Faecalibacterium prausnitzii* and correlations between reduced microbial diversity and elevated TNFα [111]. These findings built on our earlier observational study demonstrating that *Phascolarctobacterium*, a microbe associated with reduced inflammation, was significantly depleted in MPN patients compared to controls, and that *Parabacteroides* abundance was associated with TNFα levels [112]. Together, our data provide converging evidence that the MPN gut microbiome is distinct, that specific species may contribute to disease-associated inflammation, and that diet is a viable therapeutic lever in this population.

We have since extended this work with a fully online study randomizing 30 MPN patients to Mediterranean or Dietary Approaches to Stop Hypertension (DASH) diets, demonstrating the feasibility of virtual dietary interventions in MPN. Compliance was notably higher in the Mediterranean arm, and symptom burden was again reduced in both groups, further reinforcing the therapeutic potential of diet in a disease where symptom reduction is a cornerstone of treatment (Fleischman et al., this edition of Nutrients). Because MPN patients, particularly those with JAK2V617F, sit at the extreme end of cardiovascular risk, they represent an ideal model population to evaluate dietary strategies that could ultimately inform management across the broader CHIP spectrum.

## 6. Conclusions: Looking to the Future of Dietary Interventions in Clonal Hematologic Disorders

**Figure 1:**
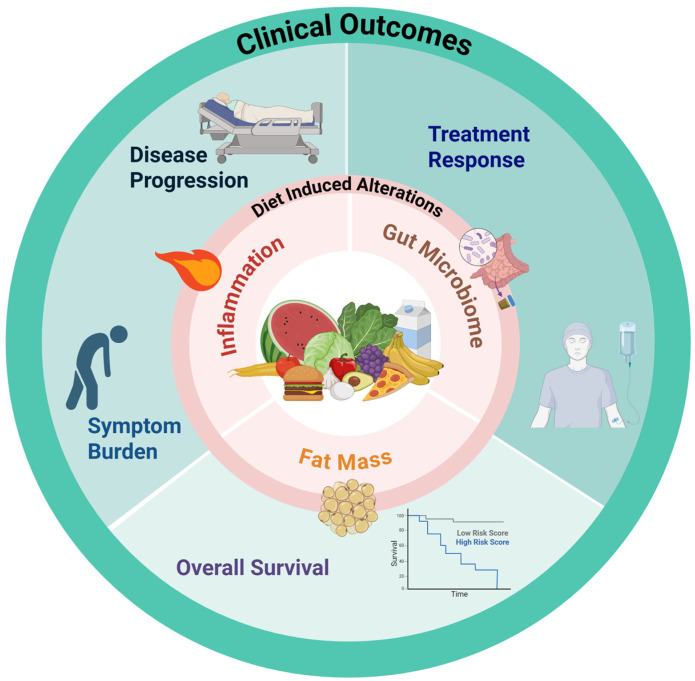
Diet influences clinical outcomes in hematologic malignancies through three interconnected biological mechanisms. Dietary patterns shape systemic inflammation, gut microbiome composition, and fat mass, each of which in turn affects disease progression, treatment response, symptom burden, and overall survival.

Despite mounting preclinical and early clinical evidence that diet can modulate inflammation, reshape the gut microbiome, and alter metabolic pathways central to clonal hematopoietic disorders, dietary intervention remains a largely untapped therapeutic strategy in this field. The evidence reviewed here demonstrates that diet has the capacity to influence disease biology at every stage of the clonal hematologic spectrum: from attenuating the inflammatory milieu that fuels CHIP expansion and its cardiovascular sequelae, to improving chemosensitivity and reducing minimal residual disease in acute leukemias, to promoting gut microbial profiles associated with sustained remission in multiple myeloma (Figure 1). That such a broadly accessible, low-toxicity intervention has received so little rigorous investigation in hematologic malignancies represents a significant gap in the field.

The therapeutic value of diet is likely distinct in each disease context, necessitating differently structured trials to evaluate its efficacy. Short-duration, high-magnitude dietary modifications such as fasting-mimicking diets administered around chemotherapy cycles are more feasible within inpatient regimens, where the short timeframe required for impact and the greater control healthcare teams have over patients’ food intake are advantageous. By contrast, demonstrating the impact of diet on long-term outcomes in CHIP is considerably more challenging, requiring large cohorts with extended follow-up. Maintaining and quantifying patient adherence to sustained dietary changes while measuring infrequent, long-term clinical events poses significant methodological difficulties. To establish the initial utility of diet in CHIP, researchers may need to rely on surrogate biomarkers that can be assessed within a shorter timeframe, such as cardiovascular risk markers, inflammatory cytokines, or changes in variant allele frequency.

Dietary interventions hold substantial potential as therapeutic adjuncts in clonal hematologic disease, but the existing evidence base is dominated by feasibility studies, observational associations, and preclinical mechanism. Recognition as routine clinical practice will require randomized controlled trials with appropriate control arms, validated endpoints, and adequate follow-up, applying the same methodological standards expected of pharmacological studies. Equally critical is the need to expand mechanistic research elucidating the molecular pathways by which dietary modifications act on clonal hematopoietic cells, the tumor microenvironment, and systemic inflammation. The convergence of accessible intervention, plausible biological mechanisms, and encouraging preliminary data creates a compelling imperative to prioritize dietary research in hematologic malignancies. Failing to do so risks overlooking one of the most promising and patient-centered avenues available for improving outcomes across the full continuum of clonal hematologic disease.

## Figures and Tables

**Table 2:** Selected dietary intervention trials and observational studies in hematologic malignancies.

Disease	Trial/Study	Intervention	*N*	Design	Key Finding
MM precursors	NUTRIVENTION (Shah 2026) [37]	High-fiber plant-based diet, 12 wk catered + 24 wk coaching	23	Single-arm	Feasible; improved BMI, insulin resistance, microbiome diversity, inflammation; 2 with improved disease trajectory
MM on lenalidomide	Shah 2022 [31]	Plant-based diet, 3 mo	Cohort	Observational	Sustained MRD negativity associated with higher stool butyrate, butyrate producers, alpha diversity
B-ALL (high risk)	IDEAL (Orgel 2021) [38]	Caloric and macronutrient restriction during induction	40	Single-arm vs. historical control	Reduced end-of-induction MRD risk; increased adiponectin; reduced insulin resistance; no fat-mass change
Pediatric ALL survivors	Zhang 2019 [100]	Family lifestyle intervention, 3 mo	13	Single-arm feasibility	Improved Healthy Eating Index; reduced added sugar; no change in physical activity or BMI
MPN	NUTRIENT (Mendez Luque 2024) [35]	Mediterranean diet vs. USDA guidelines, 15 wk	28	Randomized phase I pilot	~80% Mediterranean adherence; 53% achieved >50% reduction in MPN-SAF TSS vs. 31% control
MPN	Virtual MPN diet study (Fleischman 2026) [36]	Mediterranean vs. DASH, online	30	Randomized feasibility	Higher compliance in Mediterranean arm; symptom reduction in both arms

## Data Availability

No new data were created or analyzed in this study.
